# Clinical and behavioral determinants of oral lesions among people living with HIV/AIDS in West Java, Indonesia: a cross-sectional study

**DOI:** 10.1186/s12903-026-09132-6

**Published:** 2026-07-03

**Authors:** Irna Sufiawati, Iin Heldayani, Astrid Widhowaty Santoso, Yovita Hartantri, Rudi Wisaksana, Lucio Souza Gonçalves

**Affiliations:** 1 Department of Oral Medicine, Faculty of Dentistry, University of Padjadjaran, Jalan Sekeloa Selatan No. 1, Bandung, West Java 40132 Indonesia; 2 Department of Immunology and Infectious Diseases, Dr. Hasan Sadikin Central General Hospital, Bandung, West Java Indonesia; 3 Oral Medicine Residency Program, Faculty of Dentistry, University of Padjadjaran, Bandung, West Java Indonesia; 4 Department of Internal Medicine, Faculty of Medicine, University of Padjadjaran, Jalan Prof. Dr. Eyckman No. 38, Bandung, West Java 40161 Indonesia; 5 Postgraduate Program in Dentistry, Faculty of Dentistry, Estácio de Sá University- IDOMED, Rio de Janeiro, Brazil

**Keywords:** Human immunodeficiency virus, Oral lesions, Determinants, Access to health care, Indonesia

## Abstract

**Background:**

Oral lesions remain common among people living with human immunodeficiency virus/acquired immunodeficiency syndrome (HIV/AIDS) and may serve as important clinical indicators of immune status and disease progression, particularly in resource-limited settings. However, data from Indonesia remains limited. This hospital-based cross-sectional study aimed to characterise the spectrum of oral lesions and identify clinical and behavioral determinants associated with their occurrence among people living with HIV/AIDS (PLWHA) attending a tertiary referral hospital in West Java, Indonesia.

**Methods:**

A total of 254 PLWHA were enrolled. Sociodemographic, behavioral, and clinical data were collected through interviews and medical records, and comprehensive intraoral examinations were performed by an oral medicine specialist. Multivariable logistic regression analyses were used to identify independent determinants of oral lesions.

**Results:**

Oral lesions were identified in 75.6% of participants, with acute pseudomembranous candidiasis (27.2%), oral hairy leukoplakia (20.1%), and recurrent aphthous stomatitis (15.1%) being the most frequent diagnoses. Significant determinants of oral lesions included age ≥ 46 years (AOR 7.71; 95% CI: 1.38–43.21), underweight body mass index (AOR 4.14; 95% CI: 1.63–10.53), poor oral hygiene (AOR 4.43; 95% CI: 1.06–18.44), smoking (AOR 3.26; 95% CI: 1.51–7.08), and advanced HIV clinical stage (AOR 3.69; 95% CI: 1.71–7.95).

**Conclusions:**

Clinical and behavioral factors play an important role in the development of oral lesions among PLWHA, underscoring the importance of integrating routine oral examinations and targeted oral health strategies into HIV care, particularly in settings with limited access to health care.

## Introduction

Over the past three decades, HIV/AIDS has remained a major global public health challenge. According to recent World Health Organization (WHO) estimates, 40.8 million people worldwide were living with HIV at the end of 2024 (37.0–45.6 million), with approximately 630,000 HIV-related deaths reported globally. In Indonesia, the HIV epidemic continues to pose a substantial health burden. National epidemiological data for 2024 estimate that there are approximately 570,000 PLWHA (520,000–620,000) in the country. Despite this high burden, HIV testing coverage remains limited, potentially contributing to delayed diagnosis and progression to advanced disease stages. HIV infection disproportionately affects males and key populations, particularly men who have sex with men (MSM). Furthermore, antiretroviral therapy (ART) coverage remains suboptimal, with only 41% (37–45%) of PLWHA estimated to be receiving treatment [1].

The oral mucosa plays a critical role in assessing the health status of PLWHA, as oral manifestations often present early in the course of infection and may reflect immune suppression [2, 3]. Studies have reported that oral lesions occur in 70–90% of PLWHA across clinical stages, with at least 24 distinct oral lesions described in the literature [4–6]. The EC-Clearinghouse and WHO categorise HIV-associated oral manifestations into strongly associated, less commonly associated, and possibly associated groups. Among the most frequent findings in PLWHA are oral candidiasis, oral hairy leukoplakia, linear gingival erythema, periodontal diseases, aphthous ulcers, Kaposi’s sarcoma, and non-Hodgkin lymphoma [3, 7, 8].

The development of oral lesions in PLWHA is influenced not only by HIV-related immunosuppression but also by a range of clinical and behavioral factors. Smoking has been associated with impaired local immune responses, alterations in the oral microbiome, and increased Candida colonization, thereby increasing susceptibility to oral mucosal lesions [9, 10]. Poor oral hygiene may promote microbial dysbiosis, chronic inflammation, and opportunistic infections, particularly in immunocompromised individuals [9, 11, 12]. In addition, undernutrition can impair both systemic and mucosal immune function, reducing resistance to oral pathogens [13]. Older age has also been linked to cumulative immune decline, comorbidities, and increased vulnerability to oral diseases [14]. These factors may contribute independently or interact with HIV-related immunosuppression to increase the occurrence and severity of oral lesions.

The occurrence and severity of oral lesions correlate closely with immunologic markers such as CD4 cell count and HIV viral load. However, behavioral and sociodemographic factors, including tobacco use, alcohol consumption, oral hygiene, age, gender, and education, also influence lesion development [4, 15, 16]. Immunosuppression compromises oral mucosal defence, while behavioral and sociodemographic factors such as smoking, poor oral hygiene, alcohol use, and poor nutritional status promote microbial colonisation, inflammation, and impaired tissue healing. Despite their clinical relevance, data on oral lesions among PLWHA in Indonesia remain limited. Indonesia presents a unique context for investigating HIV-associated oral lesions because of substantial disparities in healthcare access across regions, variations in HIV testing coverage, and uneven availability of specialized oral healthcare services. West Java, one of the most densely populated provinces in Indonesia, continues to face challenges related to delayed HIV diagnosis, suboptimal retention in care, and incomplete antiretroviral therapy coverage, all of which may contribute to advanced disease presentation and a higher prevalence of HIV-associated oral lesions. These contextual factors may influence the occurrence and clinical presentation of oral manifestations and underscore the need for region-specific evidence to support integrated HIV and oral healthcare services.

This study aimed to characterize the spectrum of oral lesions and identify clinical and behavioral determinants associated with their occurrence among PLWHA attending a tertiary referral hospital in West Java, Indonesia. Determinants assessed included age, gender, education level, body mass index (BMI), smoking, alcohol use, oral hygiene, HIV clinical stage, total lymphocyte count (TLC), and ART status.

## Materials and methods

### Study design and participants

This hospital-based cross-sectional study was conducted at Dr. Hasan Sadikin General Hospital, a major tertiary referral hospital in West Java, Indonesia, between January 2022 and July 2025. The hospital receives patients from multiple districts and cities across the province, providing a diverse clinical population representative of a provincial referral setting.

The minimum required sample size was estimated using a sample size formula based on Fisher’s z transformation for testing the Pearson correlation coefficient, assuming a two-sided significance level of 5% (Zα = 1.96), a statistical power of 80% (Zβ = 0.84), and an expected correlation coefficient (r) of 0.40. Based on this calculation, the minimum sample size required was 47 participants.

Participants were recruited using a consecutive sampling method. All eligible PLWHA attending the HIV clinic during the study period who met the inclusion criteria were consecutively enrolled. The final sample consisted of 254 participants, which substantially exceeded the minimum required sample size.

The inclusion criteria were PLWHA aged 18 years or older who underwent dental and oral examinations at the study hospital during the study period and had complete medical record data for all study variables. The exclusion criteria were duplicate medical records and records with unclear or illegible information.

All participants provided written informed consent before dental and oral examinations. Clinical examinations were performed using standard diagnostic instruments under appropriate clinical conditions. Oral hygiene status was assessed using the Oral Hygiene Index-Simplified (OHI-S), which is routinely used and recorded in the clinical records at our institution. Oral hygiene was categorised as good (0.0–1.2), moderate (1.3–3.0), or poor (3.1–6.0) according to established OHI-S [17]. All oral examinations were performed by a single Oral Medicine Specialist using standardized clinical procedures. The diagnosis of oral lesions was established according to the classification system for HIV-associated oral lesions proposed by the EC-Clearinghouse and WHO to ensure diagnostic consistency.

### Data collection

Medical, demographic, behavioral, and clinical data were recorded using a standardized data collection form. Demographic variables included age, sex, education level, and BMI. Behavioral factors assessed comprised smoking status, alcohol use, and oral hygiene status as determined by the OHI-S. Clinical parameters included HIV clinical stage, TLC, and ART status.

### Data analysis

Data analysis was conducted in three stages. First, univariate analysis was performed to summarise participant characteristics using frequencies and percentages. Second, bivariate analysis using the Chi-square test was conducted to assess associations between each independent variable and the presence of oral lesions. Variables with a p-value < 0.05 in the bivariate analysis were subsequently entered into a multivariable logistic regression model. The multivariable model was used to identify independent determinants of oral lesions while adjusting for other variables. Results are presented as adjusted odds ratios (AORs) and 95% confidence intervals (CIs).

All statistical analyses were performed using IBM SPSS Statistics for Windows, Version 25.0 (IBM Corp., Armonk, NY, USA). Multicollinearity was assessed using the variance inflation factor (VIF). Model goodness-of-fit was evaluated using the Hosmer–Lemeshow test, with a p-value > 0.05 indicating an acceptable fit.

## Results

A total of 254 PLWHA were included in the analysis. Oral lesions were identified in 75.6% of participants, who were predominantly male (84.9%), with most aged 18–30 years (51.6%). A majority had a middle level of education (64.6%), normal body mass index (55.2%), were smokers (79.7%), and exhibited poor oral hygiene (72.9%). Clinically, most participants were classified as having advanced HIV disease (41.7%), had total lymphocyte counts ≥ 1200 cells/mm³ (57.3%), and had not received antiretroviral therapy (51.6%).

Bivariate analysis demonstrated significant associations between the presence of oral lesions and several variables, including male sex (*p* = 0.029), underweight BMI (*p* < 0.001), smoking (*p* < 0.001), alcohol use (*p* = 0.004), poor oral hygiene (*p* < 0.001), advanced HIV clinical stage (*p* < 0.001), and TLC < 1200 cells/mm³ (*p* < 0.001) (Table 1).

**Table 1:** Distribution of demographic, behavioral, and clinical characteristics associated with oral lesions among PLWHA (*n* = 254)

Variable	With oral lesions *n* (%)	Without oral lesions *n* (%)	*p*-value
Age			
18–30 years	99 (51.6)	34 (54.8)	0.357
31–45 years	73 (38.0)	26 (41.9)	
46–59 years	17 (8.9)	2 (3.2)	
> 59 years	3 (1.6)	0 (0)	
Gender			
Male	163 (84.9)	45 (72.6)	0.029*
Female	29 (15.1)	17 (27.4)	
Education			
Low	9 (4.7)	4 (6.5)	0.690
Middle	124 (64.6)	42 (67.7)	
High	59 (30.7)	16 (25.8)	
Body Mass Index			
Underweight	84 (43.8)	10 (16.1)	< 0.001*
Normal	106 (55.2)	46 (74.2)	
Pre-Obesity	2 (1.0)	6 (9.7)	
Smoking			
Yes	153 (79.7)	30 (48.4)	< 0.001*
No	39 (20.3)	32 (51.6)	
Alcohol Use			
Yes	72 (37.5)	11 (17.7)	0.004*
No	120 (62.5)	51 (82.3)	
Oral Hygiene			
Good	5 (2.6)	10 (16.1)	< 0.001*
Moderate	47 (24.5)	39 (62.9)	
Poor	140 (72.9)	13 (21.0)	
HIV clinical stage			
Stage 1	17 (8.9)	25 (40.3)	< 0.001*
Stage 2	38 (19.8)	18 (29.0)	
Stage 3	57 (29.7)	11 (17.7)	
Stage 4	80 (41.7)	8 (12.9)	
Total lymphocyte count			
< 1200 cells/mm^3^	82 (42.7)	11 (17.7)	< 0.001*
≥ 1200 cells/mm^3^	110 (57.3)	51 (82.3)	
ART			
Yes	93 (48.4)	36 (58.1)	0.187
No	99 (51.6)	26 (41.9)	

*N* = 254. Chi-square test was used

*OR* Odds ratio, *HIV* Human immunodeficiency virus, *TLC* Total lymphocyte count, *ART* Antiretroviral therapy

*Significant *p* < 0.05

Among the 192 participants with oral lesions, 109 (56.8%) had a single lesion, whereas 83 (43.2%) had multiple simultaneous oral lesions. Specifically, 62 participants (32.3%) had two lesions, 19 (9.9%) had three lesions, and 2 (1.0%) had four lesions. The most frequent oral lesion was acute pseudomembranous candidiasis (27.2%), followed by OHL (20.1%) and RAS (15.1%). The least frequently observed lesions (0.7% each) were gingival ulcer, Kaposi’s sarcoma, necrotizing ulcerative gingivitis, necrotizing ulcerative periodontitis, and oral herpes zoster (Table 2).

**Table 2:** Distribution of oral lesion types among HIV/AIDS patients

No.	Oral Lesion Type	*n* (%) (*N* = 298 lesions)
1	Acute pseudomembranous candidiasis	81 (27.20%)
2	Oral hairy leukoplakia (OHL)	60 (20.10%)
3	Recurrent aphthous stomatitis (RAS)	45 (15.10%)
4	Candida-associated oral lesions	24 (8.10%)
5	Linear gingival erythema (LGE)	23 (7.70%)
6	Herpes simplex virus infection	16 (5.40%)
7	Erythematous candidiasis	14 (4.70%)
8	Xerostomia	6 (2.00%)
9	Cytomegalovirus-associated ulcer	6 (2.00%)
10	Drug-related erythema multiforme	5 (1.70%)
11	Traumatic ulcer	4 (1.30%)
12	Secondary Syphilis-related oral mucous patches	4 (1.30%)
13	Gingival ulcer	2 (0.70%)
14	Kaposi’s sarcoma	2 (0.70%)
15	Necrotizing ulcerative gingivitis (NUG)	2 (0.70%)
16	Necrotizing ulcerative periodontitis (NUP)	2 (0.70%)
17	Oral herpes zoster	2 (0.70%)

Patients may present with multiple lesion types

Variables that were statistically significant in bivariate analysis were subsequently entered into a multivariable logistic regression model. The final model demonstrated good fit (Hosmer–Lemeshow test, *p* > 0.05) and no evidence of multicollinearity (all variance inflation factor values < 10). Multivariable analysis identified age ≥ 46 years (AOR 7.71; 95% CI: 1.38–43.21), underweight body mass index (AOR 4.14; 95% CI: 1.63–10.53), smoking (AOR 3.26; 95% CI: 1.51–7.08), poor oral hygiene (AOR 4.43; 95% CI: 1.06–18.44), and advanced HIV clinical stage (stages 3–4) (AOR 3.69; 95% CI: 1.71–7.95) as independent determinants of oral lesions (Table 3). Alcohol use, TLC, and ART status were not independently associated with oral lesions in the adjusted model.

**Table 3:** Independent predictors of oral lesions among PLWHA

Variable	Adjusted Odds Ratio (AOR)	95% Confidence Interval (CI)	*p*-value
Age ≥ 46 years	7.71	1.38–43.21	0.020*
Underweight BMI	4.14	1.63–10.53	0.003*
Smoking	3.26	1.51–7.08	0.003*
Poor oral hygiene	4.43	1.06–18.44	0.041*
Advanced HIV stage (3–4)	3.69	1.71–7.95	< 0.001*

Variables included in the model were those with *p* < 0.05 in bivariate analysis. The model demonstrated good fit (Hosmer-Lemeshow test, *p* > 0.05) and no multicollinearity (all VIF < 10). The model demonstrated an appropriate events-per-variable (EPV) ratio. With 192 participants presenting oral lesions and eight variables included, the EPV was approximately 24, exceeding the recommended minimum for logistic regression analyses

*Significant *p* < 0.05

## Discussion

This study found that 75.6% of PLWHA in West Java had oral lesions, a higher rate than those reported in Brazil, China, India, and Italy [18–21]. This difference may be due to variations in clinical characteristics, disease severity, access to antiretroviral therapy, healthcare services, and study settings. Many participants in this study had advanced HIV and had not begun antiretroviral therapy, which could explain the high number of oral lesions. These results highlight the need for regular oral check-ups as part of HIV care. However, oral health assessment is not consistently integrated into routine HIV services in many healthcare settings. Strengthening collaboration between HIV care providers and oral health professionals may facilitate earlier recognition of oral manifestations, improve timely referral, and support more comprehensive patient management.

Multivariable analysis identified several independent determinants of oral lesions, including advanced HIV clinical stage, poor oral hygiene, underweight body mass index, smoking, and older age. Advanced HIV disease (clinical stages 3–4) showed a strong association with the presence of oral lesions, consistent with previous studies demonstrating increased oral morbidity with progressive immune suppression and higher viral load [3, 20, 21]. However, because CD4 cell count and HIV viral load data were not consistently available in the present study, these limitations should be considered when interpreting this association. Oral candidiasis and oral hairy leukoplakia (OHL), which were the most frequently observed lesions in this study, are well-recognized markers of advanced HIV infection and have long been included among the oral lesions strongly associated with HIV infection according to the EC-Clearinghouse /WHO classification [3, 7, 8].

Recurrent aphthous stomatitis (RAS) was also among the most frequently observed oral lesions in the present study. However, unlike oral candidiasis and OHL, RAS is not classified as an oral lesion strongly associated with HIV infection according to the EC-Clearinghouse/WHO classification. Because RAS is also commonly encountered in the general population, its presence should not be considered specific evidence of HIV-related immunosuppression. Nevertheless, HIV-associated immune dysregulation may contribute to an increased frequency, severity, or persistence of aphthous lesions in susceptible individuals. Therefore, although RAS was frequently observed in this study population, it should be interpreted as a non-specific oral finding rather than a lesion uniquely associated with HIV infection.

Poor oral hygiene emerged as a significant determinant of oral lesions, supporting previous studies demonstrating that inadequate oral hygiene contributes to microbial dysbiosis, chronic oral inflammation, and increased susceptibility to opportunistic infections among immunocompromised individuals [9, 11, 12]. Alterations in the oral microbiota associated with HIV infection have been reported and are thought to play an important role in the pathogenesis of HIV-related oral diseases [9, 22]. Disruption of the oral microbial balance may enhance the virulence and adhesion of *Candida albicans*, thereby increasing the risk of oral candidiasis and other mucosal lesions [9, 23]. Although some studies have reported no association between oral hygiene and HIV-related oral lesions [21], the present findings are in line with the majority of evidence indicating a strong link between oral hygiene status and oral disease burden among PLWHA [9, 11, 12].

Smoking was also independently associated with the presence of oral lesions, in agreement with previous reports demonstrating that tobacco use alters local immune responses and facilitates fungal colonization in the oral cavity, regardless of CD4 cell count [10]. In contrast, alcohol consumption was not independently associated with oral lesions in this study, suggesting that its effect may be mediated through other behavioral or clinical factors. Previous studies have reported associations between smoking, alcohol use, and the development of oral pseudomembranous candidiasis in HIV-positive patients, although findings remain inconsistent across populations [3, 10, 21].

Nutritional status, reflected by underweight body mass index, was also identified as a significant determinant of oral lesions. Malnutrition may exacerbate immune dysfunction and impair mucosal integrity, thereby increasing susceptibility to opportunistic infections and other oral manifestations among PLWHA [13]. This effect may be further compounded by other behavioral factors identified in the present study, particularly poor oral hygiene and smoking, which can promote microbial colonization, chronic inflammation, and delayed tissue healing. Together, these factors may contribute to an increased burden of oral lesions among individuals with HIV infection. Older age was likewise associated with a higher risk of oral lesions, potentially reflecting cumulative immune decline, comorbid conditions, and longer duration of HIV infection [14]. Although age ≥ 46 years and poor oral hygiene were significantly associated with oral lesions, their corresponding confidence intervals were relatively wide, indicating limited precision of the effect estimates. This may be attributable to the relatively small number of participants in these subgroups. Therefore, these associations should be interpreted with caution and confirmed in larger studies.

Total lymphocyte count has been proposed as a surrogate marker for CD4 cell count in resource-limited settings when CD4 cell counts are unavailable [24, 25]. However, in the present study, TLC was not independently associated with oral lesions. This finding is consistent with previous reports suggesting that TLC has limited sensitivity and specificity as a predictor of oral manifestations once other clinical variables are considered [26]. Similarly, ART status was not significantly associated with oral lesions in the adjusted model. This may reflect delayed initiation of therapy, inconsistent adherence, or advanced disease at presentation. Previous studies have shown that the advent of highly active antiretroviral therapy (HAART) significantly reduces the prevalence of oral lesions by improving immune function and suppressing viral replication [6, 27]. However, some oral issues may still occur, either as adverse effects of antiretroviral therapy or because of other contributing factors, such as poor oral hygiene and smoking.

Disruptions may partially explain the high prevalence of oral lesions observed in this study, particularly regarding healthcare access during and after the COVID-19 pandemic. Reduced availability of HIV testing, delayed initiation of ART, and interruptions in routine follow-up have been reported globally and may contribute to more advanced disease presentation and a greater burden of HIV-associated oral manifestations in some patients [2, 28–30]. These findings highlight the importance of timely access to healthcare for early detection and management of oral lesions among PLWHA. In addition to their clinical impact, HIV-associated oral lesions have been shown to adversely affect oral health-related quality of life [31]. Taken together, our findings support the integration of routine oral examinations and preventive oral healthcare into HIV services, particularly in low- and middle-income countries where disparities in healthcare access and delayed HIV diagnosis remain important challenges.

This study has several limitations. Its single-centre, hospital-based cross-sectional design may limit generalizability to broader community settings and may introduce selection bias because participants were recruited from a tertiary referral centre.

Additionally, CD4 cell count and HIV viral load data were not consistently available for all participants because these investigations were not routinely documented in patients’ medical records during the study period. Consequently, alternative clinical markers, including HIV clinical stage and total lymphocyte count, were used to assess disease status. The absence of these immunological parameters limited our ability to evaluate the relationship between immune status and oral lesions directly. Therefore, the association between HIV clinical stage and oral lesions observed in this study should be interpreted with caution, as HIV clinical stage may not fully reflect the underlying immunological status of PLWHA.

Information on HIV diagnosis status (newly diagnosed versus previously diagnosed) was also not consistently available and, therefore, could not be analysed. Consequently, we were unable to determine whether diagnosis status influenced the prevalence or pattern of oral lesions observed in this study. As newly diagnosed individuals may exhibit different oral lesion profiles and levels of immunosuppression compared with patients receiving long-term HIV care, future studies should further investigate this relationship using the EC-Clearinghouse/WHO classification of HIV-associated oral lesions.

In addition, smoking and alcohol use were assessed only as binary variables based on routinely collected medical record data. Information regarding smoking duration, smoking intensity, alcohol consumption frequency, and cumulative exposure was not available. Consequently, the present study was unable to evaluate potential dose–response relationships between these behavioral factors and oral lesions, and residual confounding cannot be completely ruled out. Future studies incorporating more detailed behavioral assessments are needed to better characterize their influence on HIV-associated oral lesions.

The relatively wide confidence intervals observed for age ≥ 46 years and poor oral hygiene indicate limited precision of these effect estimates, likely due to the relatively small number of participants in these subgroups. Although the multivariable model demonstrated an adequate events-per-variable ratio, these associations should nevertheless be interpreted with caution and confirmed in larger studies. Despite these limitations, the study provides valuable insights into the burden and clinical and behavioral determinants of oral lesions among PLWHA in a tertiary referral setting in Indonesia.

## Conclusion

Oral lesions remain highly prevalent among PLWHA in West Java, Indonesia. Both HIV-related disease severity and modifiable behavioral factors were associated with the occurrence of oral lesions. These findings highlight the importance of integrating routine oral examinations and targeted preventive strategies into HIV care, particularly in resource-limited settings, to promote early detection of oral disease and improve patient management.

## Data Availability

The datasets used and/or analyzed during the current study are available from the corresponding author on reasonable request.

## Abbreviations

AIDS: Acquired immunodeficiency syndrome
AOR: Adjusted odds ratio
ART: Antiretroviral therapy
BMI: Body mass index
CI: Confidence interval
COVID-19: Coronavirus disease 2019
HAART: Highly active antiretroviral therapy
HIV: Human immunodeficiency virus
LGE: Linear gingival erythema
NUG: Necrotizing ulcerative gingivitis
NUP: Necrotizing ulcerative periodontitis
OHI-S: Oral hygiene index-simplified
OHL: Oral hairy leukoplakia
PLWHA: People living with HIV/AIDS
RAS: Recurrent aphthous stomatitis
TLC: Total lymphocyte count
WHO: World Health Organization
